# Pattern of Chromosomal Aberrations in Patients from North East Iran

**Published:** 2013-08-24

**Authors:** Saeedeh Ghazaey, Farzaneh Mirzaei, Mitra Ahadian, Fatemeh Keifi, Tootian Semiramis, Mohammad Reza Abbaszadegan

**Affiliations:** 1 Pardis Clinical and Genetics Laboratory, Mashhad, Iran; 2 Division of Human Genetics, Immunology Research Center, Avicenna Research Institute, Mashhad University of Medical Sciences, Mashhad, Iran; 3 Medical Genetic Research Center, School of Medicine, Mashhad University of Medical Sciences, Mashhad, Iran

**Keywords:** Chromosomal Aberrations, Cytogenetic Analysis, North East Iran

## Abstract

**Objective:**

Chromosomal aberrations are common causes of multiple anomaly syndromes. Recurrent chromosomal aberrations have been identified by conventional cytogenetic methods used widely as one of the most important clinical diagnostic techniques.

**Materials and Methods:**

In this retrospective study, the incidences of chromosomal aberrations were evaluated in a six year period from 2005 to 2011 in Pardis Clinical and Genetics Laboratory on patients referred to from Mashhad and other cities in Khorasan province. Karyotyping was performed on 3728 patients suspected of having chromosomal abnormalities.

**Results:**

The frequencies of the different types of chromosomal abnormalities were determined, and the relative frequencies were calculated in each group. Among these patients, 83.3% had normal karyotypes with no aberrations. The overall incidences of chromosomal abnormalities were 16.7% including sex and autosomal chromosomal anomalies. Of those, 75.1 % showed autosomal chromosomal aberrations. Down syndrome (DS) was the most prevalent autosomal aberration in the patients (77.1%). Pericentric inversion of chromosome 9 was seen in 5% of patients. This inversion was prevalent in patients with recurrent spontaneous abortion (RSA). Sex chromosomal aberrations were observed in 24.9% of abnormal patients of which 61% had Turner’s syndrome and 33.5% had Klinefelter’s syndrome.

**Conclusion:**

According to the current study, the pattern of chromosomal aberrations in North East of Iran demonstrates the importance of cytogenetic evaluation in patients who show clinical abnormalities. These findings provide a reason for preparing a local cytogenetic data bank to enhance genetic counseling of families who require this service.

## Introduction

cal cytogenetics began its rapid advancement with the discovery of the correct 46 chromosomes in human in 1956 (1). After that, various types of major chromosomal syndromes with modified numbers of chromosomes such as Down syndrome (trisomy 21), Turner’s syndrome (45, X) and Klinefelter’s syndrome (47, XXY) were detected (1). About 1000 chromosomal abnormalities have been identified to this date. This makes a major contribution to human morbidity and mortality (2). The common human disorders including mental retardation, congenital malformation, sterility, sexual abnormalities and spontaneous fetal loss may be due to chromosomal abnormalities (3). The majority of abnormal fetuses are spontaneously aborted and the prevalence is 0.6% in live births (4). Congenital abnormalities with chromosomal defects cause gross phenotypic anomalies and are the main causes of mental retardation (5, 6). The disorders of sexual development associated with abnormal sex chromosome karyotypes are Turner’s syndrome, Klinefelter’s syndrome, certain menstrual disorders (primary amenorrhea and secondary amenorrhea), Superman syndrome, true and pseudohermaphroditisms.

Genetic counseling services are important sources of information about the incidence of chromosomal aberrations. These centers allow to detect the types or profiles of chromosomal aberrations and also to establish the pattern of chromosomal variability in distinct populations. Increased awareness about chromosomal abnormalities among physicians has resulted in an increase in identification of many chromosomal disorders. The aim of the present cytogenetic evaluation was to find out the incidence of different chromosomal abnormalities in patients from North East of Iran. We determined the most common cytogenetic abnormalities at the Cytogenetic Department of the Pardis Clinical and Genetics Laboratory (PCGL) in Mashhad, Iran. Additionally, the frequencies of chromosomal abnormalities were calculated for comparison with data reported in similar previous studies.

## Materials and Methods

In this retrospective study, over a six year period (2005-2011), 3728 cases were attended by the Genetic Counseling Services in PCGL or by practicing physicians of a wide variety of specialties. For preservation of samples and cytogenetic analysis, all cases gave informed consent that was approved by the Ethics Committee of Mashhad University of Medical Sciences.

All the referral cases were thoroughly examined and detailed clinical and family histories were recorded. The cases were classified according to the reasons for referral. For routine cytogenetic analysis, 5 ml peripheral blood samples were collected into heparinized test tubes. Short term lymphocyte cultures were set up according to Moorhead et al. (7). 400 µl blood cells were cultured in 5 ml RPMI 1640 (GIBCO, USA), supplemented by 20% (v/v) fetal bovine serum (FBS, GIBCO, USA), and 10 μg/ml phytohemagglutinin (GIBCO, USA) at 37˚C for 72 hours. Metaphases were harvesthed by adding colcemid (GIBCO, USA) for 10 minutes followed by hypotonic KCl treatment (Merck, Germany) for 15 minutes and fixation using standard 3:1 methanol-acetic acid fixative (Merck, Germany). The karyotypes were determined by G-banding using trypsin and Giemsa (GTG) (8) and C-banding using barium and Giemsa (CBG) when necessary (9). Wellbanded metaphases were photographed using iAi photomicroscope (iAi, Japan) and were analyzed by Cytovision software (Applied Imaging, USA) at 400-550 band resolution. At least 30 metaphases were routinely analyzed. In cases of mosaicism, 100 metaphase spreads were examined. The best metaphases were photographed to specify the karyotypes. Karyotype analyses were carried out according to the International System for Human Cytogenetics Nomenclature (ISCN 2009) (10). Statistical analyses were carried out by comparing the correlation between age and chromosomal abnormalities, using SPSS version 16.

## Results

After clinical examinations, the patients investigated were grouped according to the clinical findings into specific diagnostic categories. 48.5% of referral cases were females and 51.5% were males. Among these, 622 cases (16.7%) showed chromosome abnormalities (Table 1). Autosomal abnormalities were found in 467 cases (75.1%) and abnormalities of the X and Y were found in 155 cases (24.9%).

 cases ranged from 18 to 45, with a mean of 33.61 (Standard Deviation (SD) =5.3). Our data revealed that there was not any significant correlation between chromosomal aberration and age (p>0.05). The majority of referral cases were couples with recurrent spontaneous abortion (RSA). The number of RSA varied from 1-7 (mean 2.93, SD=1.29). The results showed the correlation between number of abortions, chromosomal aberrations and couple’s age was significant (p0.05) in this category. Both husband and wife were examined and only 3.4% of patients showed abnormal karyotypes. These were 7.7% of our total abnormal cases. Of these cases, 52.1% had balanced reciprocal translocations. Robertsonian translocations were seen in 10.4% abnormal patients. According to karyotyping analysis, there was a significant correlation between translocation and RSA (p0.05). Of the remaining cases, 37.5% showed pericentric inversions in chromosomes 9(p11q13) and Y (p11q11.2), although inv (9) has long been regarded a normal variant (11) (Table 2). The next most common alterations were Down syndrome, intellectual disability, dysmorphic features, congenital anomalies, and developmental delay, Turner’s and Klinefelter’s syndromes (Table 1).

Of the 291 cases referred for mental retardation, dysmorphic features, congenital anomalies and developmental delay, 44 cases (15.1%) did not show normal karyotypes. These abnormalities included 19 trisomies, 13 inversions (although inv (9) has long been regarded a normal variant (11)), one unbalanced translocation, one duplication, eight deletions and two ring chromosomes. One of the carriers of ring chromosomes is a patient with mosaicism of deletion and monosomy. The details are summarized in table 3. After karyotyping, the patients were grouped according to their karyotyping results into three categories: (a) normal karyotype, (b) autosomal abnormalities and (c) sex chromosome abnormalities.

**Table 1:** Distributionof chromosomal aberrations according to clinical features


Reason for referral (Categories)	Abnormal		Total	
	%(In category)	No	%	No

**Turner’s syndrome**	25.1	63	6.7	251
**Primary or secondary amenorrhea**	13.3	56	11.3	421
**Klinefelter’s syndrome**	32.7	35	2.9	107
**Infertility**	6.5	31	12.9	480
**Ambiguous genitalia**	11.7	15	3.4	128
**Down syndrome**	57.1	330	15.5	578
**Mental retardation/dysmorphic features/congenital anomalies/ developmental delays and other abnormalities**	15.1	44	7.8	291
**Recurrent spontaneous abortions**	3.4	48	38.1	1422
**Consanguineous marriages**	0	0	1.3	50
**Total**	16.7	622		3728


**Table 2:** Cytogenetic results for couples with recurrent spontaneous abortion


Karyotype	No

**d reciprocal translocations**	25
**Robertsonian translocations**	5
**Inversions**	18
**Total**	48 (7.7%)


**Table 3:** Cytogenetic results for cases referred for diagnosis of mental retardation, dysmorphic features, congenital anomalies and developmental delays


Karyotype	No

**47, XX, +21**	5
**47, XY, +21**	7
**47, XX, +13**	3
**46, XY, t (13; 14) (q10; q10)**	1
**47, XY, +8/46, XY**	2
**47, XY, +18**	1
**47, XX, +18**	1
**46, XY, r (18) (p11.32q32)**	1
**46, XX, r (18) (p11.32q22.1)/46, XX, del (18) (q22.1)/45, XX, -18**	1
**46, XX, del (18) (p11.1)**	3
**46, XY, del (18) (p10)**	2
**46, XY, del (5) (p15.1)**	2
**46, XX, del (5) (p15.1)**	1
46, XX, inv (9) (p11q13)*	5
46, XY, inv (9) (p11q13)*	8
**46, XX, dup (2) (p12p13)**	1
**Total**	44 (7.1%)


t; Translocation, r; Ring chromosome, del; Deletion, inv; Inversion, and dup; Duplication. *; This inversion is normal variation.

### Normal karyotypes

Out of 3728 cases, 3106 karyotypes were normal (46, XX or 46, XY). However, these patients were suspected of chromosomal aberrations according to their clinical features such as RSA, infertility or mental retardation. Of 128 cases for ambiguous genitalia and sex determination, only 15 cases (11.7%) showed sex reversal. Karyotypes of these patients were structurally and numerically normal, thus were classified in the normal category. However, the success of providing an accurate laboratory diagnosis for these cases needs to be improved using fluorescence in situ hybridization and other complementary molecular approaches.

### Autosomal abnormalities

Down syndrome (DS), observed in 360 cases (77.1%), was the most common autosomal abnormality with the highest frequency among total abnormal results (57.9%). Five cases had mosaicism (1.4%) and 18 cases (5%) inherited DS due to translocation of chromosome 21 to 21 or chromosome 14 to 21. Among the DS patients, there were three cases (0.8%) with trisomy chromosome 21 and inversion in chromosome 9 (Table 4). Among abnormal cases, there were four cases of Patau’s syndrome with full trisomy 13 (0.9%) and two infants with Edward’s syndrome (full trisomy 18) (0.4%). According to our data, we could not see any mosaicism and the age was less than 10 days. Part of our prospective study, they died before reaching one month. Among abnormal cases, three patients had cri-du chat syndrome (0.6%) with de novo deletion in the short arm of chromosome 5. Parents of these infants were analyzed to determine whether one of them carried a reciprocal translocation or had mosaicism with normal and 5p-cells. Since there were no chromosomal abnormalities in the parents, the risk of recurrence in future siblings greatly reduces. 

In other structural autosomal aberrations, translocations were the most common abnormality seen in 47 cases (66.2%) followed by deletions in 10 cases (14.1%), inversions in seven cases (9.9%), ring chromosomes in five cases (7%) and an addition and a duplication were seen in two separated cases (1.4%; Table 4).

 inversion of chromosome 9 was seen in 31 patients (6.6%) where 27 cases (5.8%) had only pericentric inversion 9 (p11q13). In addition, there were three patients with inv (9) accompanied with trisomy 21 and one case accompanied with Klinefelter’s syndrome. Although inv (9) has long been regarded a normal variant (11) but according to their abnormal karyotyping results, it was classified in this group.

**Table 4:** Autosomal abnormalities found by karyotyping


Cases	Karyotype	No	%

**Down syndrome**		360	77.1
	47, XX (Y) +21	334	
	47, XX (Y) +21/46, XX (Y)	5	
	46, XX (Y), t (14; 21) (q10; q10), +21	8	
	46, XX (Y),t (21; 21) (q10; q10), +21	10	
	47, XX (Y), inv(9) (p11q13), +21	3	
**Patau’s syndrome**		4	0.9
	47, XX, +13	3	
	46, XY, t (13; 14) (q10; q10)	1	
**Cri-du-chat syndrome**		3	0.6
	46, XY, del5 (p15.2)	2	
	46, XX, del (p15.2)	1	
**Edward’s syndrome**	47, XX, +18	2	0.4
**Inv (9)**	46, XX (Y), inv(9) (p11q13)*	27	5.8
**Other aberrations**	-	71	15.2
**Total**		467	75.1


t; Translocation, inv; Inversion and del; Deletion. *; This inversion is normal variation.

### Sex chromosome abnormalities

Turner’s syndrome was the most common sex chromosome abnormality among diagnosed cases. In 94 Turner’s patients, the most frequent result was the classic karyotype (45, X) (52.1%). The majority of other Turner’s showed mosaicism with isochromosome X (i), isodicentric X (idic), triple X, ring chromosome X (r) and inversion X and a Y chromosome component (45, X/46, XY) in their karyotyping. Among Turner’s patients, 17 cases had isochromosome X (18.1%) and, three of them had isodicentric chromosome X (3.2%). Among women with other sexual abnormalities, two cases had three X chromosomes (1.3%) and one was mosaicism with two cell lines of (47, XXX/46, XX) (0.65%). The most frequent sex chromosome abnormality in our male group was Klinefelter’s syndrome. There were 52 abnormal cases (33.5%). Inversion of Y chromosome was observed in five cases (p11q11.2) (3.2%). Only one man had superman syndrome with 47, XYY result (0.65%). The details of sex chromosome aberrations are summarized in table 5.

**Table 5:** Sex chromosomal abnormalities found by karyotyping


Cases	Karyotype	No	%

**Turner’s syndrome**		94	61
	45, X	49	
	45, X/46, XX	5	
	45, X/46, XY	4	
	45, X/46, X, i (Xq)	6	
	45, X/46, X, i (Xq)/46, XX	1	
	45, X/46, X, idic (Xq)	3	
	47, XXX/45, X/46, XX	3	
	45, X/46, X, r (X) (q21.3q23)	2	
	45, X/46, X, add (X) (q22.3q24)	1	
	45, X/47, X, i (X) (q10)x2/46, X, i(X) (q10)	1	
	45, X/46, X, inv (X) (p21.2p22.3)	1	
	45, X/46, X, del (X) (p22.1q22)	1	
	47, XYY/45, X/46, XY	1	
	44, X, t (15; 21)/45, X, i (Xq), t (15; 21)	1	
	46, X, i Xq)	8	
	46, X, del (Xq)	7	
**Trisomy X**		3	1.9
	47, XXX	2	
	47, XXX/46, XX	1	
**Klinefelter’s syndrome**		52	33.5
	47, XXY	50	
	46, XXY, t (13; 14) (q10; q10)	1	
	47, XXY, inv (9) (p11q13)	1	
	47, XYY		
**Superman syndrome**		1	0.6
**Other abrrerations**	46, X, inv (Y) (p11q11.2)	5	3.2
**Total**		155	24.9


t; Translocation, r; Ring chromosome, del; Deletion, inv; Inversion, i; Isochromosome, idic; Isodicentric and add; Addition.

**Table 6:** Comparison of the results of present study with that of Balkan et al. (2010) and Akbari et al. (1998)


Reason for referral	Akbari et al. (1998)				Balkan M et al. (2010)				Current study			
(Categories)	Abnormal		Total		Abnormal		Total		Abnormal		Total	

	%	No	%	No	%	No	%	No	%	No	%	No
**Turner’s syndrome**	14	7	13.3	51	19.6	95	8.5	486	25.1	63	6.7	251		
**Primary or secondary amenorrhea**	0	0	1.6	6	16.4	56	6.0	342	13.3	56	11.2	421
**Klinefelter’s syndrome**	27	14	13.6	52	25.3	92	6.4	364	32.7	35	2.8	107
**Ambiguous genitalia**	27	6	5.7	22	13.6	22	2.9	162	11.7	15	3.4	128
**Down syndrome**	86	26	7.8	30	53.2	557	18.4	1048	57.1	330	15.5	578
**Mental retardation/dysmorphic features/ congenital anomalies/ developmental delay**	15	3	3.9	15	5.3	30	10.0	568	15.1	44	7.8	291
**Repeated abortions**	1.7	3	44.4	170	2.3	44	33.3	1892	3.4	48	38.1	1422


## Discussion

The current study aimed to evaluate the pattern of chromosomal aberration incidence in North East of Iran. In addition, this study compared the distribution of chromosomal aberrations in this area with other similar reports performed in Iran by Akbari et al. (12) and in Turkey by Balkan et al. (13). We chose Turkey because of the similarity of Iran and Turkey in religion, geography and demography. In our study, the total prevalence of chromosomal aberrations (16.7%) was noticeably higher than that was reported in several studies in general population (0.5-0.6%) (14-16). Consequently, it was higher than 12% which was reported by Khalil et al. (17), lower than 29.3% which was reported by Duarte et al. (4) and similar to 16.1 and 16.5% which was reported by Balkan et al. and Akbari et al. respectively (12, 13).

Furthermore, we analyzed the frequency of chromosomal abnormalities in each category. The main reason of referrals for cytogenetic studies was RSA. In our study the prevalence of carriers of chromosomal abnormalities among them was 6.8% per couple. This incidence was reported as 4.6% in Turkey (13), 3.5% in previous report from Iran (12) and 7.4% in Saudi Arabia (18). The majority of investigated couples had normal karyotypes but abnormal results were due to balanced translocations that carriers usually are clinically normal with increased risk of producing offspring with unbalanced translocation. Risk for unbalanced gametes depends on the location of chromosome break points relative to the centromere and cross-over frequency (19). The majority of our patients with inv (9) karyotype were cases with RSA (23%). Although the inv (9) is a variant of normal karyotype, it was not possible to confirm whether inv (9) was responsible for these clinical features; however, it can result in infertility and recurrent abortion as it can act on acentric fragments formed in meiosis and synaptonemal complexes respectively (20). The second prevalent cause of referrals was Down syndrome. Chromosome 21 trisomy is the most frequent aneuploidy in human populations. It occurs in 1:700 newborns (21). In our study males with Down syndrome accounted for 59% of the Down cases. A similar gender ratio is observed by Balkan et al. (13) as 53.1% males. In contrast, there was considerable variability between our study and previously reported study in Iran that the great percentage was in favor of females with DS (12). In addition, our results revealed that the majority of investigated DS individuals (92.8%) were born due to the classic karyotype with only the extra free chromosome 21. This finding was comparable with 88% of Iran DS patients (12) and 88.7% of Turkey DS patients (13).

The third clinical feature in the current report was the presence of mental retardation and dysmorphic feature. The majority of these patients had normal karyotypes. The prevalence of abnormal cases in this category (15.1%) was very similar to the previous study in Iran (15%) (12) but was higher than that (5.3%) reported by Balkan et al. (13). We revealed that the prevalent anomalies in this category were trisomy 18 and 13 (0.05% and 0.1% respectively) which were roughly similar to Turkey’s study (0.07 and 0.03% respectively). Since the majority of fetuses with Edward’s syndrome are spontaneously aborted, it is not common to see patients with this disease (4, 22).

The other aspect of this study was the determination of sex chromosome aberrations which showed that Turner’s syndrome was the most frequent sex chromosome abnormality (61%). Turner’s syndrome afflicts approximately 1 in 2000 females and is the most common factor in infertile women (23). The distribution of Turner’s syndrome and amenorrhea in the category were nearly similar in the present study (25.1 and 13.3% respectively) and the Balkan study (13) (19.6 and 16.4% respectively) but were different from Akbari’s report (12) (14 and 0% respectively). 

The second frequent sex chromosome aberration was Klinefelter’s syndrome being more frequent in infertile males (23). Two groups of males were referred to us for cytogenetic study: adult males with infertility and subfertility problems and juvenile males with microtestis. In general, the findings of this study are in accordance with most investigations which confirm the XXY aneuploidy to be the most prevalent chromosomal aberration in infertile male (2-5, 12, 13, 17, 19). In our study, 32.7% of men assumed to have Klinefelter’s syndrome had abnormal karyotype. This was similar to previous studies in Iran (27%) (12) and Turkey (25.3%) (13). 

The last category among our patients was consanguineous marriages. Consanguineous marriages are traditionally favored in most Asian and African populations especially in Muslim countries. However, it is apparent that these types of marriages are an important element for some autosomal recessive genetic disorders (24). Although the frequency of consanguineous marriages was 1.3% in our study and lower than the Balkan report (6%) (13), no chromosomal abnormality was seen in people referred for consanguineous marriages.

## Conclusion

The present data is the first report from the Northeastern region of Iran, a single clinical service; therefore it could not represent the prevalence of chromosomal aberrations in the Iranian population as a whole. In this regard, more cytogenetic studies are needed. In addition, we need complementary molecular tests to improve the results. However we hope that the data gathered by such reports will provide a basis for diagnostic purposes, the risk assessment of genetic disease recurrences and for clinical treatment decision making, management and better genetic counseling.

## References

[1] Smeets DF (2004). Historical prospective of human cytogenetics: from microscope to microarray. Clin Biochem.

[2] Goud MT, Al-Harassi SM, Al-Khalili SA, Al-Salmani KK, Al-Busaidy SM, Rajab A (2005). Incidence of chromosome abnormalities in the Sultanate of Oman. Saudi Med J.

[3] Shah VC, Murthy DS, Murthey SK (1990). Cytogenetic studies in a population suspected to have chromosomal abnormalities. Indian J Pediatr.

[4] Duarte AC, Cunha E, Roth JM, Ferreira FL, Garcias GL, Martino-Roth MG (2004). Cytogenetics of genetic counseling patients in Pelotas, Rio Grande do Sul, Brazil. Genet Mol Res.

[5] Pashayan H, Dallaire L, MacLeod P (1973). Bilateral aniridia, multiple webs and severe mental retardation in a 47, XXY- 48, XXXY mosaic. Clin Genet.

[6] Kour A, Mahajan S, Singh JR (2003). Cytogenetic profile of individuals with mental retardation. Int J Hum Genet.

[7] Moorhead PS, Nowell PC, Mellman WJ, Battips DM, Hungerford DA (1960). Chromosome preparation of leukocytes cultured from human peripheral blood. Exp Cell Res.

[8] Seabright M (1971). A rapid banding technique for human chromosomes. Lancet.

[9] Salamanca F, Armendares S (1974). C bands in human metaphase chromosomes treated by bariumhydroxide. Ann Genet.

[10] Shaffer LG, Slovak ML, Lynda J, Campbell LJ (2009). An international system for human cytogenetic nomenclature.

[11] Yamada K (1992). Population studies of INV (9) chromosomes in 4,300 Japanese: incidence, sex difference and clinical significance. Jpn J Hum Genet.

[12] Akbari MT, Behjati F, Ashtiani Khaleghian M (1998). Chromosomal abnormalities in a referred population: a report of 383 Iranian cases. Acta medica Iranica.

[13] Balkan M, Akbas H, Isi H, Oral D, Turkyilmaz A, Kalkanli S (2010). Cytogenetic analysis of 4216 patients referred for suspected chromosomal abnormalities in Southeast Turkey. Genet Mol Res.

[14] Hamerton JL, Canning N, Ray M, Smith S (1975). A cytogenetic survey of 14,069 newborn infants.I.Incidence of chromosome abnormalities. Clin Genet.

[15] Hook EB, Hamerton JL, Hook EB, Porter IH (1977). The frequency of chromosome abnormalities detected in consecutive newborn studies; differences between studies; results by sex and by severity of phenotypic involvement. Population cytogenetics: studies in humans.

[16] Patil SR, Lubs HA, Brown J, Cohen M, Gerald P, Hecht F (1977). Incidence of major chromosome abnormalities in children. Cytogenet Cell Genet.

[17] Khalil MS, Badr FM, Al Saidi K (2007). Pattern of chromosomal abnormalities among referral cases in a tertiary health care facility in Riyadh, Saudi Arabia. Suez Canal Univ Med J.

[18] Al Husain M, Zakib OK (1999). A survey of 1,000 cases referred for cytogenetic study to King Khalid University Hospital, Saudi Arabia. Hum Hered.

[19] Luthardt FW, Keitges E Chromosomal syndromes and
genetic disease Encyclopedia of life science. Nature Publishing Group.

[20] Mozdarani H, Mohseni Meybodi A, Zari-Moradi S (2008). A cytogenetic study of couples with recurrent spontaneous abortions and infertile patients with recurrent IVF/ICSI failure. Indian J Hum Genet.

[21] Dursun P, Gultekin M, Yuce K, Ayhan A (2006). What is the underlying cause of aneuploidy associated with increasing maternal age? Is it associated with elevated levels of gonadotropins?. Med Hypotheses.

[22] Giaccardi A, Sardi R, Priora U (1991). Vivalda, M, Domeneghetti G, Girone P.Trisomy 18 or Edwards’ syndrome.A report of 4 clinical cases. Minerva Pediatr.

[23] Salahshourifar I, Sadat Masoudi N, Gourabi H (2009). Cytogenetic findings in couples who are candidates for assisted reproductive techniques. Yakhteh.

[24] Hasanzadeh-Nazarabadi M, Rezaeetalab GH, Dastfan F (2006). Study of youths’ knowledge, behavior, and attitude towards consanguineous marriages. Iranian J Public Health.

